# Penile Rehabilitation After Surgery for Prostate Cancer: An Umbrella Review on Traditional Approaches and Novel Perspectives

**DOI:** 10.3390/jcm15124688

**Published:** 2026-06-17

**Authors:** Giuseppe Seminara, Leonardo Meduri, Marco Leuzzi, Gabriele Antonini, Antonio Aversa

**Affiliations:** 1 Department of Experimental and Clinical Medicine, Magna Graecia University, 88100 Catanzaro, Italy; dott.giuseppeseminara@gmail.com; 2 Endocrinology Unit, Renato Dulbecco University Hospital, 88100 Catanzaro, Italy; leonardo.meduri@studenti.unicz.it (L.M.); marco.leuzzi2@studenti.unicz.it (M.L.); 3 Antonini Urology, 00196 Rome, Italy; gabrieleantoninimd@gmail.com

**Keywords:** radical prostatectomy, erectile dysfunction, penile rehabilitation, PDE5 inhibitors, vacuum erectile device, penile prosthesis, platelet-rich plasma, low-intensity extracorporeal shockwave therapy, regenerative medicine

## Abstract

**Background/Objectives**: Penile rehabilitation (PR) techniques are claimed to counteract chronic degenerative processes of cavernous tissue, such as penile hypoxia, neurovascular damage, and cavernous fibrosis. The objective of this umbrella review is to synthesize findings from existing meta-analyses to evaluate the efficacy of traditional and emerging PR strategies, providing an evidence-based roadmap for clinical management after surgery for prostate cancer. **Methods**: Conducted in accordance with PRIOR guidelines, a comprehensive literature search of PubMed, the Cochrane Library, and Scopus was performed through April 2026. The review included primarily systematic reviews and meta-analyses investigating pharmacological, physical, surgical, and regenerative interventions for post-prostatectomy erectile dysfunction (ED). Methodological quality was independently assessed using standardized tools. **Results**: PDE5 inhibitors (PDE5-is) significantly improve erectile function during active treatment, yet evidence supporting their role in promoting spontaneous, “unassisted” recovery remains limited. Vacuum erectile devices demonstrate high efficacy for assisted intercourse but show minimal impact on returning to baseline function compared to placebo. Penile prosthesis (PP) implantation maintains robust efficacy with exceptionally high satisfaction rates (83–85%), proving independent of prior pelvic surgery. Although early-phase trials suggest clinical potential for regenerative therapies like low-intensity extracorporeal shockwave therapy, platelet-rich plasma, and stem cell interventions, the evidence is currently undermined by substantial heterogeneity in study protocols and concerns regarding methodological quality. **Conclusions**: PR following radical prostatectomy remains a complex challenge characterized by poor evidence. While PDE5-Is are established first-line therapy for assisted function, PP remains the most reliable definitive treatment for refractory ED cases. Regenerative approaches show promise but remain investigational until standardized protocols and large-scale trials are established.

## 1. Introduction

Prostate cancer continues to represent a formidable global health challenge, ranking as the second most common malignancy and the fifth leading cause of cancer-related mortality among men worldwide [1]. With the continued refinement of screening programs and the aging global population, the volume of definitive treatments—specifically radical prostatectomy (RP)—has reached unprecedented levels [2]. Despite the widespread adoption of minimally invasive technologies, most notably robot-assisted radical prostatectomy (RARP), and the meticulous anatomical descriptions of the neurovascular bundles, post-surgical functional outcomes remain a critical concern [3]. While oncological control and urinary continence have seen significant improvements, the preservation and recovery of erectile function (EF) remain the most elusive goals in prostate cancer surgery [4].

The burden of iatrogenic erectile dysfunction (ED) extends far beyond the loss of penetrative capacity; it permeates the psychological, social, and relational fabric of the patient’s life [5]. For many men, the sudden loss of EF is perceived as a “de-masculinizing” event, frequently leading to profound shifts in masculine identity, decreased self-esteem, and a heightened risk of clinical depression and anxiety [5]. Furthermore, sexual dysfunction often causes significant distress for the partner and a withdrawal from intimacy, which can destabilize long-term relationships during an already stressful period of cancer survivorship [5]. This psychosocial burden is compounded by the chronic nature of the condition, as the failure to achieve early recovery can lead to “sexual abandonment,” where patients and couples cease all sexual activity, further complicating later rehabilitative efforts [5].

From a pathophysiological perspective, the rationale for intervention is rooted in the prevention of irreversible structural changes. The trauma to the cavernous nerves, whether through direct injury, traction, or thermal damage, induces a state of chronic penile hypoxia and overexpression of pro-fibrotic growth factors [6]. This oxygen deprivation triggers the pro-fibrotic cascade, characterized by the apoptosis of cavernous smooth muscle cells and the subsequent deposition of collagen within the corpora cavernosa [6]. This remodeling leads to venous leak (corporeal veno-occlusive dysfunction), often rendering the erectile tissue refractory to conventional pharmacological therapies if left untreated [7].

The concept of “penile rehabilitation” (PR) was introduced to counteract this degenerative process, shifting the clinical paradigm from a reactive, “wait-and-see” approach to a proactive, multi-modal strategy aimed at preserving tissue integrity during the window of nerve recovery [8]. Over the past two decades, the therapeutic armamentarium has expanded from the foundational use of phosphodiesterase type 5 inhibitors (PDE5i) and vacuum erectile devices (VED) to advanced intracavernosal therapies and, more recently, to the frontiers of regenerative medicine, including low-intensity extracorporeal shockwave therapy (Li-ESWT) and stem cell (SC)-based interventions [9].

However, the rapid proliferation of PR protocols has not been matched by a consensus on the most effective strategy. The existing literature is characterized by a high volume of primary studies and systematic reviews with often discordant findings, heterogeneous outcome measures, and varying levels of methodological rigor. This fragmentation of evidence poses a significant challenge for clinicians in providing evidence-based counseling. Consequently, there is an urgent need for a high-level synthesis to distill the current state of the art. This umbrella review aims to evaluate the quality and findings of existing studies—prioritizing meta-analytical findings—to provide a comprehensive, expert-level roadmap for the management of post-prostatectomy ED.

## 2. Materials and Methods

This umbrella review was conducted in accordance with the preferred reporting items for overviews of reviews (PRIOR) guidelines [10]. The review protocol was registered on the PROSPERO database (registration number: CRD420261372308).

The study was designed according to the population, intervention, comparison, outcome (PICO) model [11]. The included population considered men affected with iatrogenic ED following any type of radical prostatectomy (i.e., open, laparoscopic, or robot-assisted), the interventions investigated were all medical, physical, and surgical therapies for ED, while the comparison included either no intervention or a placebo or other treatments. Primary and secondary outcomes were differently chosen and ranked based on clinical relevance.

A comprehensive literature review was conducted in April 2026. The search strategy included the following keywords: ‘prostatectomy’, ‘radical prostatectomy’, ‘robotic-assisted radical prostatectomy’, ‘RARP’, ‘nerve-sparing prostatectomy’, ‘prostate cancer’, ‘PCa’, ‘iatrogenic erectile dysfunction’, ‘penile rehabilitation’, ‘erectile function recovery’, ‘sexual health recovery’, ‘PDE5 inhibitors’, ‘PDE5i’, ‘phosphodiesterase type 5 inhibitors’, ‘sildenafil’, ‘tadalafil’, ‘vardenafil’, ‘avanafil’, ‘intracavernosal injections’, ‘ICI’, ‘alprostadil’, ‘combination therapy’, ‘vacuum erectile device’, ‘VED’, ‘penile prosthesis’, ‘penile implant’, ‘inflatable penile prosthesis’, ‘IPP’, ‘low-intensity extracorporeal shockwave therapy’, ‘Li-ESWT’, ‘shockwave therapy’, ‘platelet-rich plasma’, ‘PRP’, ‘stem cell therapy’, and ‘regenerative medicine’ in PubMed, the Cochrane Library and Scopus. In addition to database searches, the reference lists of all included studies and relevant previous reviews were manually screened to identify further pertinent articles. The search was updated before the final analysis to ensure the inclusion of the most recent evidence.

Study eligibility included human subjects, English language publication, and no limitations on publication date. Systematic reviews of randomized controlled trials (RCTs) with meta-analyses of individual or cumulative data were selectively reported. Systematic reviews with meta-analyses of prospective non-randomized or cohort studies or without data synthesis (including only qualitative analysis) that used systematic, structured, and reproducible methods to collect data from primary research were also analyzed when systematic reviews of RCTs with meta-analyses were not intercepted. The most recent and highest quality thematic review was chosen if multiple systematic reviews evaluated the same intervention. In the absence of meta-analytical data, primary research was included according to a hierarchy of evidence, prioritizing RCTs, followed by non-RCTs, and cohort studies. Furthermore, such primary studies were included even if they had not been captured by recent existing meta-analyses. If multiple systematic reviews evaluated the same intervention, the most recent and highest quality thematic review was chosen. Duplicative systematic reviews were only included if they were comparable in quality and published in the same year or presented a clear conclusion.

The literature searches, data extraction, and tabulation were carried out independently by three reviewers (G.S., L.M., and M.L.) and verified by two independent reviewers (G.A. and A.A.). The quality of the studies was assessed using the Methodological Quality of Systematic Reviews 2 [AMSTAR-2, http://www.amstar.ca (accessed on 16 May 2026)] [12] for systematic reviews, revised tool for risk of bias (rRoB 2, https://www.riskofbias.info/welcome/rob-2-0-tool, accessed on 16 May 2026) [13] for RCTs, risk of bias in non-randomized studies of intervention (ROBINS-I) [14] for prospective non-randomized studies, and the Newcastle–Ottawa scale [http://www.ohri.ca/programs/clinical_epidemiology/oxford.asp, (accessed on 16 May 2026)] [15] for observational/cohort studies. Three reviewers (G.S., L.M., and M.L.) independently assessed the methodological quality of all included studies. Additionally, the certainty of evidence for primary outcomes was directly extracted from the formal GRADE (grading of recommendations, assessment, development, and evaluations) assessments conducted within the highest-quality included systematic reviews, rather than performing a de novo grading, to prevent compounding bias resulting from primary study overlap.

A qualitative synthesis was performed using aggregate data as reported in the original publications.

## 3. Results

Three relevant scientific areas were identified regarding PR treatments: 1. Conventional medical therapy; 2. Surgical therapy; 3. Regenerative therapy.

### 3.1. Conventional Medical Therapy

#### 3.1.1. PDE5-is

The efficacy of PDE5-is in PR following RP has been extensively evaluated across several meta-analyses, yielding the following core findings.

The most recent meta-analysis of 39 RCTs found that PDE5-is are significantly superior to a placebo in improving EF during the treatment period, as measured by the International Index of Erectile Function–Erectile Function (IIEF-EF) domain scores [16]. In particular, compared with the placebo group, patients in the regular PDE5-i group (mean difference (MD): 3.07; 95% confidence interval (CI): 1.69 to 4.44; *p* < 0.0001; I^2^: 96%) and on-demand group (MD: 3.92; 95% CI: 2.95 to 4.88; *p* < 0.00001; I^2^: 99%) had a significantly higher mean IIEF-EF scores within 3 months after RP [16]. These results were even more significant in patients undergoing regular PDE5-i therapy for more than 6 months after RP (MD: 4.70; 95% CI: 3.66 to 5.74; *p* < 0.00001; I^2^: 0%) [16]. As for the proportion of IIEF-EF ≥ 22, patients in the regular group and the on-demand PDE5-i group had a significantly higher proportion than those in placebo group 6 months after RP, and the odds ratios (OR)s were 1.87 (95% CI: 1.32 to 2.66; *p* = 0.0005; I^2^: 11%) and 2.17 (95% CI: 1.20 to 3.93; *p* = 0.01; I^2^: 51%), respectively [16].

The debate regarding the optimal administration protocol—regular daily or nightly versus on demand—is a central focus. No significant difference was observed between daily and on-demand PDE5-i groups regardless of mean IIEF-EF score (*p* = 0.75) or the proportion of IIEF-EF ≥ 22 (*p* = 0.61) in the long term [16].

Moreover, a critical distinction remains between “assisted” and “unassisted” recovery. In a meta-analysis of 4 RCTs, when the data after PDE5-i washout were analyzed, the result showed that PDE5-i did not improve spontaneous EF (OR: 1.027; 95% CI: 0.713 to 1.478; *p* = 0.610; I^2^: 48.4%) [17]. The Cochrane Review (2018) emphasizes that there is no high-certainty evidence demonstrating that scheduled (nightly or daily) use leads to superior long-term recovery of unassisted (spontaneous) EF [18]. In particular, treatment discontinuation was not significantly different after at least 6 months of daily PDE5-i therapy compared with a placebo [relative risk (RR): 1.12; 95% CI: 0.85 to 1.48; *p* = 0.41] or on-demand therapy (RR: 1.09; 95% CI: 0.86 to 1.38; *p* = 0.98; I^2^: 0%) [18]. Otherwise, in a network meta-analysis of 22 RCTs involving 2711 patients, which compared different interventions for PR after nerve-sparing RP, only the daily dose of sildenafil 100 mg reached statistical significance for unassisted EF recovery (OR 4.00; 95% CI 1.40 to 13.4) [19].

Furthermore, early initiation of therapy post-RP is generally advocated to prevent cavernous fibrosis and structural changes. A prospective RCT demonstrated that immediate initiation of sildenafil after RARP significantly improved EF recovery at 12 months compared to delayed treatment [41.4% vs. 17.7%; hazard ratio (HR): 2.943; *p* = 0.034] [20]. However, the “curative” potential of early intervention remains under scrutiny due to the low to very low certainty of long-term evidence in high-quality RCTs [18].

PDE5-i are globally recognized as safe and well-tolerated in the post-prostatectomy population. The most common adverse events identified across meta-analyses include headache [Peto odds ratio (POR): 2.99; 95% CI: 2.22 to 4.04; *p* < 0.00001; I^2^: 0%], dyspepsia (POR 3.15; 95% CI: 1.86 to 5.35; *p* < 0.0001; I^2^: 0%), flushing (POR 4.71; 95% CI: 3.19 to 6.95; *p* < 0.00001; I^2^: 0%), and nasal congestion (POR 2.66; 95% CI: 1.85 to 3.84; *p* < 0.00001; I^2^: 0%) [21]. In the short term, the risk of serious adverse events (defined according to the National Cancer Institute Common Terminology Criteria for Adverse Events [22]; grades 3 to 5) was rare but higher in patients undergoing on-demand therapy compared to a placebo (RR: 0.32; 95% CI 0.11 to 0.94; *p* = 0.04; I^2^: 0%) [18]. No differences were found between the risk of serious adverse events comparing on-demand and daily PDE5-i therapy in the short term (RR: 0.69; 95% CI: 0.12 to 4.04; *p* = 0.68) and long term (RR: 3; 95% CI: 0.13 to 71.92) [18].

A summary of the main quantitative findings on PDE5-i therapy for PR has been summarized in Table 1.

#### 3.1.2. VEDs

The efficacy of VEDs in PR following RP has been evaluated across several systematic reviews and meta-analyses.

The most recent meta-analysis of RCTs found that International Index of Erectile Function-5 items (IIEF-5) scores were significantly higher in the VED group compared to controls at 3 months (MD: 9.70; 95% CI: 5.16 to 14.24; *p* < 0.0001) and 6–9 months after RP (MD: 6.70, 95% CI: 2.30 to 11.10; *p* = 0.003; I^2^: 72%) [16]. A recent meta-analysis of two RCTs reported a VED efficacy rate of 84.5% (95% CI: 73–96%) for successful intercourse or intercourse satisfaction specifically in post-RP patients [23].

Otherwise, the network meta-analysis by Sari Motlagh et al. (2021), which analyzed 22 RCTs, did not identify VED among the interventions with a significantly higher likelihood of EF recovery (i.e., the proportion of patients who experienced a return to the baseline EF at the end of washout) compared to the placebo (OR 1.59, 95% CI 0.53 to 5.24) [19]. This suggests that while VED is effective for assisted sexual activity, its role as a standalone rehabilitation strategy for spontaneous EF recovery may be limited.

Notably, a systematic review of seven RCTs, five prospective and four retrospective observational studies, identified considerable heterogeneity in VED protocols across studies [24]. Among 16 included studies, seven used daily VED protocols, while rehabilitation duration varied: less than 1 year in four studies, up to 12 months in six studies, and more than 1 year in six studies [24].

VEDs are generally well-tolerated with transient and non-serious adverse events. The meta-analysis by Zhang et al. reported that the most common side effect was penile bruising, with a pooled incidence of 25.3% (95% CI: 0.18 to 0.30); however, no sub-analysis was made in relation to VED use for PR after RP [23].

#### 3.1.3. Prostaglandins

Evidence about prostaglandin use for PR is limited. The efficacy of an intracavernosal alprostadil injection for PR was evaluated in a single RCT involving 30 patients with preoperative good erections after nerve-sparing RP [25]. In particular, patients randomized to alprostadil injections three times per week (for 12 weeks), noted 67% recovery of spontaneous erection after the 6-month follow-up (in comparison with 20% in the observation group, *p* < 0.01) [25].

The Cochrane Review (2018) included a direct comparison of daily PDE5-is versus daily intraurethral prostaglandin E1 for PR [18]. Based on very low-quality evidence derived from a single RCT [26], efficacy was comparable between the two regimens, with no significant difference in EF recovery (RR: 1.1; 95% CI: 0.79 to 1.52; *p* = 0.59) [18].

However, the most recent high-quality systematic reviews with meta-analysis found no eligible trials comparing scheduled intracavernosal alprostadil injections versus on-demand use for PR, representing a significant gap in the evidence base [16,18].

No quantitative analysis was found concerning the adverse effects of prostaglandins in the context of PR. A recent systematic review and meta-analysis, which included 12 studies (5 RCTs and 7 non-RCTs), compared the adverse effects of intracavernosal injection therapy using isolated and combined substances [27]. Priapism showed a minimal presence in the isolated alprostadil group (OR: 1.84, 95% CI: 1.03 to 3.20, *p* = 0.04); however, when analyzing only RCTs, there was no significant difference between groups [27]. Fibrosis rates ranged from 0.8% to 15% across studies [27].

#### 3.1.4. Pelvic Floor Muscle Training (PFMT)

PFMT is proposed as a physiotherapy intervention for improving EF after RP. While pooled data from three RCTs involving 126 patients indicated no significant improvement in favor of PFMT compared to control group at 3–6 months postoperatively (OR: 2.37; 95% CI: 0.79 to 7.07; *p* = 0.12; I^2^: 0%) [16], a recent network meta-analysis of 22 RCTs found a significantly higher likelihood of EF recovery with PFMT (OR: 5.21; 95% CI: 1.24 to 2.98), but with a low certainty of evidence due to risk of bias assessment and small sample size (n = 51) of two RCTs [19].

### 3.2. Surgical Therapy

Penile prosthesis (PP) is not properly considered a PR strategy, since it does not aim to rehabilitate penile function. However, when patients with post-RP ED fail to achieve adequate recovery through conventional medical interventions, surgical intervention becomes the definitive treatment strategy. Consequently, PP implantation is recommended as the third-line therapy for patients who are refractory to or have contraindications for non-invasive therapies. Our search yielded no results regarding systematic reviews with meta-analyses specifically dedicated to the use of PP in treating patients following prostate surgery. However, as this treatment is symptomatic and independent of the underlying cause of ED, efficacy outcomes from the general ED population can be generalized and considered comparable to the subcategory of iatrogenic post-prostatectomy ED.

The most recent and comprehensive meta-analysis, incorporating 83 studies (32 prospective and 51 retrospective) and 12,132 subjects with a mean follow-up of 47.6 months, demonstrated an exceptionally high overall patient satisfaction rate of 83% (95% CI: 80.6 to 86.3) following PP implantation [28]. When comparing device types, satisfaction rates were significantly higher for inflatable penile prostheses (IPP) (85.4%; 95% CI: 81.1 to 88.8) compared to malleable (72.4%; 95% CI: 63.2 to 80) or semi-rigid (84.5%; 95% CI: 81.1 to 87.3) devices, with three-piece IPPs yielding the highest levels of patient satisfaction [28]. More specifically, PP implantation significantly improved the combination of IIEF-5 and IIEF-EF scores (SMD: 5.73; 95% CI: 4.51 to 6.95), after pooling data from 14 studies involving 1263 patients [28]. Interestingly, meta-regression analyses indicated that patient satisfaction is independent of the presence of pelvic surgery (as RP) or trauma (S: 0.003; 95% CI: −0.001 to 0.009; *p* = 0.102) [28].

From a safety perspective, long-term complications associated with PP implantation are limited [28]. The mean incidence of mechanical failure is reported at approximately 4.6%, while device erosion occurs in roughly 3% of cases [28]. Post-operative infection rates remain low, ranging between 2.9% and 4.5%; however, the occurrence of infectious complications is tightly correlated with the presence of diabetes mellitus [28].

### 3.3. Regenerative Therapy

#### 3.3.1. Li-ESWT

Li-ESWT has recently been considered as a treatment for ED, and more specifically for PR after prostate surgery. A meta-analysis was conducted specifically evaluating Li-ESWT efficacy for PR [29]. Li-ESWT significantly improved International Index of Erectile Function (IIEF) score after 3–4 months in the pooled analysis of three RCTs (n = 200) (MD: −2.04; 95% CI: −3.72 to −0.35; *p* = 0.02) and after 9–12 months in a single RCT (n = 85) (MD: −1.80; 95% CI: −2.54 to −1.06; *p* < 0.00001) [29]. However, heterogeneity between studies was high (73%) due to consistently different treatment protocols [29]. In particular, higher energy flux density ranged from 0.09 to 0.15 mJ/mm^2^, total pulses for each treatment ranged from 1500 to 4000, and pulses for each region from 300 to 600 [29].

A recent systematic review with meta-analysis compared the benefit of Li-ESWT–PDE5-i association with monotherapy for EF [30]. In the sub-analysis related to treatment of EF caused by RP (2 RCTs, n = 109), IIEF-5 score improvement was not significantly different when comparing Li-ESWT and PDE5-i association with PDE5-i or prostaglandin monotherapy [standard mean difference (SMD): 0.26; 95% CI: −1.12 to 1.65; *p* < 0.01; I^2^: 91%] [30].

No meta-analytic findings were identified concerning adverse events of Li-ESWT after RP. However, the most recent high-quality meta-analysis on Li-ESWT therapy for ED found no treatment-related adverse events in the short term when pooling data from 20 RCTs (n = 1400) [risk difference (RD): 0.0; 95% CI: −0.01 to 0.02; *p* = 0.73; I^2^: 0%] [31].

#### 3.3.2. Platelet-Rich Plasma (PRP)

Our search did not identify any meta-analyses or individual RCTs regarding the use of PRP for ED following prostate surgery, reflecting the limited evidence available for this still-emerging methodology. The most recent high-quality meta-analysis on PRP monotherapy of mild-to-moderate ED of any cause intercepted six RCTs (n = 441) comparing this intervention versus placebo [32]. Interestingly, statistically non-significant results on IIEF scores improvement were found when pooling data at 1 month (SMD: 1.06; 95% CI: −0.38 to 2.50; I^2^: 85%), 3 months (SMD: 1.01; 95% CI: −0.40 to 2.42; I^2^: 84%) and 6 months of follow-up (SMD: 1.43; 95% CI: −0.22 to 3.08; I^2^: 89%) [32]. The high heterogeneity across studies reflected the different treatment protocols applied across studies (i.e., PRP injection volumes ranged from 5 to 10 mL in 2–4 sessions) [32]. Adverse events were infrequently reported, and only one study described single cases of hematoma or plaque formation [32].

A different meta-analysis evaluated the efficacy of PRP in combination with Li-ESWT [33]. The pooled analysis of seven RCTs comparing PRP alone or in association with Li-ESWT vs. placebo or Li-ESWT monotherapy showed a significant improvement in the IIEF score in the experimental group only after 24 weeks of follow-up (MD 2.46; 95% CI: 1.10 to 3.83; *p* = 0.0004; I^2^: 86%) [33]. Otherwise, peak systolic velocity (PSV) was significantly augmented after 4 weeks (1 RCT, n = 100) (MD: 14.50; 95% CI: 12.46 to 16.54; *p* < 0.00001), 12 weeks (1 RCT, n = 113) (MD: 8.40; 95% CI: 4.96 to 11.84; *p* < 0.00001), and 24 weeks (2 RCTs, n = 213) (MD: 13.75; 95% CI: 11.57 to 15.93; *p* < 0.00001; I^2^: 32%) [33]. Moreover, after pooling data from three RCTs (n = 357), the association between PRP and Li-ESWT significantly improved the IIEF score at 24 weeks of follow-up compared to Li-ESWT monotherapy (MD: 2.57; 95% CI: 1.34 to 3.80; *p* < 0.0001; I^2^: 66%) [33].

#### 3.3.3. Stem Cells (SCs)

Among the most recent therapies proposed for regenerative purposes to repair nerve damage following RP is the use of intracavernous SCs of various origins. No systematic reviews with meta-analyses focusing specifically on this patient population were found in the literature. The most recent meta-analysis on the use of SCs for ED in men aggregates several clinical trials (RCTs or uncontrolled trials) involving patients with ED from various causes, including post-RP cases [34]. The results of the pooled data indicate a significant increase in PSV after three months (SMD: 0.63; 95% CI: 0.28 to 0.98; *p* < 0.0001; I^2^: 46.31%) and six months (SMD: 1.2; 95% CI: 0.23 to 2.19; *p* = 0.015; I^2^: 71.24) [34]. Two studies found a significant improvement in post-therapy IIEF-5 scores at three months (SMD: 1.05; 95% CI: 0.61 to 1.49; *p* < 0.0001; I^2^: 50.16%) and six months (SMD: 1.23; 95% CI: 0.77 to 1.70; *p* < 0.0001; I^2^: 51.59%) [34]. Otherwise, the pooled analysis from two studies did not find statistically significant differences in IIEF scores three months after SC therapy (SMD: −0.11; 95% CI: –0.58 to 0.36; *p* = 0.647; I^2^ < 0.0001), while a little improvement was found at six months (SMD: 0.64; 95% CI: 0.13 to 1.15; *p* = 0.014; I^2^ < 0.0001) [34]. Finally, two studies evaluated the baseline and post-therapy six-month IIEF-EF scores, indicating a significant improvement in this outcome (SMD: 1.57; 95% CI: 0.98 to 2.16; *p* < 0.0001; I^2^ < 0.0001) [34]. Sources of heterogeneity included differences in SC type (i.e., bone marrow, adipose tissue, Wharton jelly, oral mucosa, dental pulp, placental matrix, and umbilical cord blood), administration protocols, and patient characteristics (i.e., diabetic, post-RP) [34]. Among the clinical trials discussed in this systematic review, four clinical trials reported no adverse effects related to SCs, while four clinical trials reported minor side effects such as minor discomfort, irritation, and minor pain, redness, swelling, local reaction, and itching at the injection site [34].

Two phase I–II trials focused on SC therapy for PR [35,36]. The first one utilized intracavernous autologous adipose-derived SCs on 17 men (age: 46–69 years), reporting that eight (47.1%) recovered their erectile function and were able to accomplish sexual intercourse [35]. Post hoc stratification according to urinary continence status was performed. Interestingly, for the continent group, 8 out of 11 men recovered EF (MD: 0.57; 95% CI: 0.38 to 0.85; *p* = 0.0069), while incontinent men did not regain erectile function (MD: 1; 95% CI: 0.85 to 1.18; *p* > 0.9999) [35]. The second study used intracavernous autologous bone marrow mononuclear cells in six patients [36]. After 6 months, significant improvements compared to the baseline were noted in IIEF–intercourse satisfaction (*p* = 0.033) and IIEF-EF (*p* = 0.035) domains [36].

### 3.4. Study Quality Assessment

The methodological quality of all the included studies in the present umbrella review is included in Table 2.

Regarding systematic reviews, most of which included a meta-analysis, the results reveal a heterogeneous landscape: six studies demonstrated high methodological quality [18,28,30,31,32,33]. Three studies [19,23,24] were judged to be of moderate quality. However, a significant portion of the included reviews [16,17,21,24,29] was found to have critically low quality, indicating potential limitations in the robustness of the evidence reported in these papers.

Both RCTs included in the review [20,25] exhibited a high risk of bias, suggesting that their clinical data should be interpreted with caution.

Finally, both phase I/II clinical trials [35,36] reported a critical risk of bias.

## 4. Discussion

The management of ED following RP remains a complex clinical challenge, requiring a shift from reactive treatments to proactive, multi-modal PR strategies. This umbrella review provides a synthesis of the current evidence, evaluating traditional pharmacological and non-pharmacological approaches alongside emerging regenerative therapies to offer a comprehensive roadmap for clinicians. While the therapeutic armamentarium has expanded significantly over the last two decades, our analysis reveals a landscape characterized by varying levels of methodological rigor and a persistent lack of consensus on the most effective protocols.

PDE5-is remains the first-line treatment, showing significant efficacy in improving EF during active treatment [22]. Evidence suggests that long-term regular use (over 6 months) yields more significant IIEF-EF score improvements compared to a placebo [22]. However, the strength of evidence regarding “unassisted” or spontaneous recovery is low, with several systematic reviews finding no significant difference in spontaneous EF after a washout period [17]. Furthermore, while early initiation is widely advocated to prevent cavernous fibrosis, its long-term curative potential remains supported by low to very low certainty evidence.

VEDs demonstrate high efficacy for assisted intercourse, with satisfaction rates reaching approximately 84.5% [23]. However, their role in facilitating spontaneous recovery is questionable, as they do not significantly increase the likelihood of returning to baseline EF compared to a placebo [21]. Similarly, evidence for PFMT is conflicting; while network meta-analyses suggest a benefit [21], the small sample sizes and high risk of bias in primary RCTs result in low certainty of evidence [16].

PP implantation represents the definitive third-line therapy for patients refractory to non-invasive treatments. The evidence supporting PP is robust, showing exceptionally high satisfaction rates (83–85%), particularly with three-piece inflatable devices [28]. Notably, meta-regression indicates that patient satisfaction is independent of a history of pelvic surgery like RP, suggesting it is a highly reliable option for this sub-population [28].

Emerging therapies such as Li-ESWT and SC interventions show promise in early-phase trials and meta-analyses. Li-ESWT has been shown to significantly improve IIEF scores at 3–12 months post-prostatectomy [29]. SC therapy also indicates potential for improving PSV and IIEF scores at 6 months [34]. However, these results are tempered by high heterogeneity in treatment protocols and a critical risk of bias in many primary studies [35,36].

PRP currently lacks specific meta-analytic evidence for the post-prostatectomy population, and general ED data shows no significant benefit for PRP monotherapy [32]. The relative lack of robust evidence and the significant heterogeneity observed across clinical studies are the primary factors behind why current guidelines continue to classify this methodology as investigational [37,38,39].

To better understand the clinical efficacy and limitations of these rehabilitation strategies, their specific biological mechanisms of action must be considered in relation to post-prostatectomy pathophysiology. Conventional medical therapies primarily target immediate hemodynamic and structural preservation. PDE5-is function by preventing the degradation of cyclic guanosine monophosphate, thereby amplifying nitric oxide-mediated cavernous smooth muscle relaxation [40]. In the post-operative setting, regular daily or nightly dosing aims to maintain chronic corporeal oxygenation, directly counteracting the tissue hypoxia that develops during cavernous nerve neuropraxia [41]. Conversely, VEDs utilize mechanical negative pressure to draw a mixture of arterial and venous blood passively into the corpora cavernosa. This mechanical engorgement stretches the corporeal tissue, a physical expansion that limits structural retraction and mitigates progressive cavernous fibrosis [42]. When the neurovascular bundles are severely damaged, prostaglandins provide a critical alternative; they bypass the compromised NO pathway entirely by binding to prostanoid receptors and stimulating adenylate cyclase to elevate intracellular cyclic adenosine monophosphate, inducing direct, nerve-independent smooth muscle relaxation [43]. Complementing these, PFMT acts as a physical therapy intervention that strengthens the bulbocavernosus and ischiocavernosus muscles, thereby optimizing the veno-occlusive mechanism and helping patients retain active blood flow within the erectile bodies. When conservative measures fail, rather than rehabilitating damaged tissue, PP structurally replaces the dysfunctional erectile tissue with inflatable or malleable cylinders, completely bypassing the damaged neurovascular and microvascular infrastructure to provide immediate rigidity independent of biological nerve recovery [44]. Finally, emerging regenerative approaches aim to alter the underlying tissue microenvironment rather than just temporarily assisting function. Li-ESWT delivers acoustic waves that cause shear stress and microtrauma within the cavernous tissue. This mechanical stress triggers the release of angiogenic factors, which improve local microvascular perfusion and recruit endogenous progenitor cells to the site of injury. In a similar regenerative vein, PRP delivers a highly concentrated pool of autologous growth factors directly into the corpora, activating cellular proliferation and tissue remodeling pathways while suppressing pro-apoptotic signaling. Lastly, SC therapies exert their therapeutic effects predominantly through paracrine signaling. The injected cells secrete a potent array of neurotrophic, immunomodulatory, and angiogenic cytokines that accelerate the repair of iatrogenic cavernous nerve injury, preserve smooth muscle cell content, and actively inhibit the pro-fibrotic signaling cascade that drives collagen deposition and subsequent venous leak.

Given these distinct biological pathways, the deployment of combination therapy has emerged as a highly logical and potentially superior strategy for penile rehabilitation. By pairing modalities with complementary mechanisms of action, clinicians can theoretically achieve synergistic effects that single-agent interventions cannot provide. For example, combining daily PDE5-is with VED therapy may ensure both biochemical support and the structural preservation of corporeal tissue elasticity during the prolonged window of neuropraxia. Similarly, associating microvascular regenerative strategies like Li-ESWT or PRP with standard pharmacological therapy aims to actively repair the underlying vascular and neural architecture while simultaneously amplifying downstream tissue compliance. Our review highlighted that while the mechanistic rationale for these combinations is robust, the translation of high-level clinical evidence remains mixed and heavily dependent on the specific sub-population evaluated. For instance, current meta-analytical data examining the combination of Li-ESWT and PDE5-is demonstrated significant efficacy in the general erectile dysfunction population, yet subgroup analyses isolated to post-prostatectomy patients failed to establish a statistically significant advantage over monotherapy [30,31]. Conversely, combining regenerative treatments, such as PRP and Li-ESWT, has shown compelling long-term improvements in erectile function scores compared to shockwave monotherapy, though data strictly dedicated to the post-surgical cohort is still limited [33]. This discrepancy underscores a critical crossroad in modern penile rehabilitation: while the combination of mechanical, biochemical, and regenerative mechanisms is pathophysiologically optimized to combat iatrogenic tissue degeneration, large-scale, protocol-standardized trials are urgently required to validate these multi-modal frameworks specifically for prostate cancer survivors.

A primary challenge identified in this review is the high heterogeneity observed across studies, particularly regarding VED protocols, Li-ESWT energy flux densities, and the origin and administration of stem cells. Methodological quality is also a concern; while six of the included systematic reviews were of high quality, a significant portion (five reviews) was judged to have critically low quality according to AMSTAR-2. Additionally, many of the primary RCTs and clinical trials in this field exhibit a high or critical risk of bias, which mandates caution when interpreting functional outcomes.

The present review has several strengths. First of all, this review utilizes an “umbrella” approach, distilling findings from multiple meta-analyses to provide the highest level of evidence currently available. Moreover, the study adhered to PRIOR guidelines and utilized standardized tools (AMSTAR-2, RoB 2, ROBINS-I) for objective quality assessment. The literature search was conducted through April 2026, ensuring the inclusion of the most recent clinical developments and meta-analyses.

Nevertheless, we must acknowledge several limitations. Many emerging therapies (PRP, SCs) lack dedicated meta-analyses for the post-prostatectomy sub-group, forcing reliance on broader ED population data. The overall strength of our conclusions is limited by the critically low quality of several underlying systematic reviews and the high risk of bias in available RCTs. Moreover, by limiting eligibility to English-language publications, some relevant international data may have been omitted. Furthermore, a baseline de novo GRADE re-evaluation was not systematically performed across all pooled data due to the inherent heterogeneity and significant primary study overlap characteristic of the umbrella review design; however, GRADE certainty levels reported by the original high-quality reference reviews were meticulously preserved and reported textually. Finally, a key inherent limitation of conducting an umbrella review is the loss of granular clinical data; the majority of the available meta-analyses pool heterogeneous patient populations without systematic stratification based on crucial variables such as the exact degree of nerve-sparing, baseline erectile function, age, or exposure to pelvic radiation, which complicates the extraction of personalized predictive outcomes.

When translating these findings into clinical practice, it is crucial to recognize that penile rehabilitation outcomes are heavily influenced by a multitude of patient-specific and perioperative variables. The literature consistently demonstrates that younger age, excellent preoperative erectile function, and bilateral nerve-sparing surgery represent the triad of highest success for spontaneous recovery and response to first-line therapies like PDE5-is [2,3,4]. Conversely, unilateral nerve-sparing significantly degrades recovery rates, while non-nerve-sparing procedures fundamentally shift the therapeutic timeline toward early third-line interventions like PP implantation. Furthermore, the introduction of perioperative or adjuvant chemoradiation adds a profound layer of microvascular and neural damage that often renders iatrogenic erectile dysfunction refractory to traditional non-invasive strategies. Beyond surgical treatment, understanding the long-term functional toll of external radiotherapy is essential for comprehensive patient counseling. Recent clinical evidence exploring moderate hypofractionated radiotherapy combined with protective optimization protocols indicates that approximately 37.5% of previously potent men experience a worsening of erectile function (de novo erectile dysfunction) at 18 months post-treatment, with older age identified as a critical predisposing factor for severe erectile decline [45]. While the transition to robot-assisted techniques has improved anatomical visualization, the biological prerequisites of the patient and the structural preservation of the neurovascular bundles remain the true drivers of rehabilitative efficacy.

The total volume of nerve-sparing procedures has significantly escalated in recent years, a clinical shift heavily propelled by earlier cancer detection strategies and the diagnostic integration of multiparametric magnetic resonance imaging and quantitative histology, which actively assist clinicians in predicting non-organ-confined disease prior to surgery [46]. Conversely, this growing emphasis on quality-of-life preservation highlights a delicate clinical paradox where, in select cases, optimal post-operative functional outcomes have arguably been prioritized over definitive oncological outcomes [47]. Furthermore, the introduction of perioperative or adjuvant chemoradiation adds a profound layer of microvascular and neural damage.

Beyond clinical efficacy and safety profiles, the economic burden associated with penile rehabilitation therapies represents a major determinant of long-term patient compliance and therapeutic adherence. Because these protocols often require prolonged or multi-modal regimens, the financial impact on the patient is highly variable and strictly dependent on geographic location, national healthcare models, and individual insurance coverage. From a relative cost perspective, first-line oral PDE5-is represents a generally low-cost, recurrent expense due to the widespread availability of affordable generic formulations, although public or private insurance coverage varies significantly by country and often imposes strict monthly pill limits. Similarly, supportive physical modalities such as VEDs and PFMT involve relatively low financial barriers; VEDs require a modest, one-time purchase of a medical-grade device, while PFMT typically requires a limited course of physical therapy sessions that are frequently integrated into standard post-operative physical rehabilitation packages. Moving to advanced therapies, intracavernosal injections represent a moderate, ongoing cost per injection, but are widely recognized and partially or fully reimbursed by healthcare systems as an established second-line therapy. Conversely, PP implantation, despite presenting a high upfront cost, serves as a definitive third-line strategy for refractory cases. In stark contrast, emerging regenerative modalities carry a high to very high financial burden for the patient. Because current international guidelines continue to classify these approaches as investigational, they are universally excluded from public and private reimbursement frameworks, remaining strictly out-of-pocket, cash-pay treatments. The results of our findings underline that clinicians should adopt a patient-centered, multi-modal approach to penile rehabilitation. PDE5-is remains the standard starting point, but patients should be informed that these may primarily assist in “assisted” function rather than a guaranteed “curative” recovery. For those seeking definitive results, early referral for PP should be considered, given the high satisfaction rates. VEDs and PFMT can be integrated as supportive therapies, though their role in long-term nerve recovery is less certain. To synthesize the clinical evidence compiled in this review into an actionable framework, Table 3 serves as a step-by-step decision flowchart guiding early multi-modal intervention based on patient stratification and treatment response.

To move the field forward, there is an urgent need to develop consensus-based protocols for Li-ESWT, PRP, and SC therapies to reduce heterogeneity. Large-scale, multicenter RCTs with long-term follow-up and standardized “unassisted” recovery measures (e.g., using a washout period) are needed. Moreover, it would be useful to investigate the synergistic effects of combining regenerative therapies (e.g., PRP plus Li-ESWT) specifically within the post-RP population. Furthermore, studies identifying predictive factors of response to individual therapies would be desirable to better guide patients toward the most effective personalized therapeutic strategy (i.e., [44,48]). Finally, research should further address the psychological and relational burden of iatrogenic ED to provide more holistic survivorship care.

## 5. Conclusions

At present, the clinical management of post-RP ED remains a complex challenge, characterized by an evolving therapeutic landscape that still lacks a unified consensus. While PDE5-Is remains the first-line treatment and penile prostheses may offer an effective definitive solution for refractory-ED patients, intermediate options such as VEDs and PFMT still present conflicting evidence regarding their ability to promote spontaneous functional recovery. Furthermore, although emerging therapies like Li-ESWT and SC interventions show promising potential, their clinical application is currently limited by significant heterogeneity in treatment protocols and variable methodological quality across primary studies. Therefore, it is crucial that future research focuses on harmonizing protocols and strengthening methodological rigor in order to define proactive, evidence-based therapeutic pathways that can truly improve long-term outcomes for prostate cancer survivors.

## Figures and Tables

**Table 1:** Summary of the main meta-analyses selected for each outcome regarding PDE5-i therapy for PR after RP.

Main Outcome	Specific Outcome	Reference	Studies Included	Patients Included	Results
IIEF-EF score improvement	Daily dose (≤3 months)	Feng et al., 2022 [16]	5 RCTs	668 (340 intervention group vs. 328 control group)	MD: 3.07; 95% CI: 1.69 to 4.44; *p* < 0.0001; I^2^: 96%
	Daily dose (≥6 months)	Feng et al., 2022 [16]	6 RCTs	261 (133 intervention group vs. 128 control group)	MD: 4.70; 95% CI: 3.66 to 5.74; *p* < 0.00001; I^2^: 0%
	On-demand dose (≤3 months)	Feng et al., 2022 [16]	4 RCTs	749 (426 intervention group vs. 323 control group)	MD: 3.92; 95% CI: 2.95 to 4.88; *p* < 0.00001; I^2^: 99%
	On-demand dose vs. daily dose (<6 months)	Feng et al., 2022 [16]	2 RCTs	159 (79 on-demand dose group vs. 80 daily dose group)	MD: 0.39; 95% CI: −2.04 to 2.82; *p* = 0.75; I^2^: 0%
	On-demand dose vs. daily dose (≥6 months)	Feng et al., 2022 [16]	2 RCTs	152 (78 on-demand dose group vs. 74 daily dose group)	MD: 0.49; 95% CI: −8.80 to 9.79; *p* = 0.92; I^2^: 92%
Spontaneous EF after treatment discontinuation	PDE5-i efficacy (all types)	Liu et al., 2017 [17]	4 RCTs	1181 (777 intervention group vs. 404 control group)	OR: 1.027; 95% CI: 0.713 to 1.478; *p* = 0.610; I^2^: 48.4%
	PDE5-i efficacy (by drug type)	Sari Motlagh et al., 2021 [19]	22 RCTs	2711 (1880 treated with interventions vs. 831 controls)	Daily sildenafil 100 mg (OR: 4.0; 95% CI: 1.40 to 13.40) Daily sildenafil flexible dose (OR: 2.61: 95% CI: 0.45 to 17.3) Daily sildenafil 25 mg (OR: 2.07; 95% CI: 0.47 to 10.4) Daily sildenafil 50 mg (OR: 1.94; 95% CI: 0.82 to 5.35) Daily vardenafil 5 mg (OR: 3.27; 95% CI: 0.49 to 2.56) Daily vardenafil 10 mg (OR: 2.22; 95% CI: 0.33 to 19.5) Vardenafil on demand (OR: 1.02; 95% CI: 0.44 to 2.36) Daily vardenafil flexible dose (OR: 0.77; 95% CI: 0.33 to 1.84) Daily tadalafil 20 mg (OR: 1.38; 95% CI: 0.38 to 51.16) Daily tadalafil 5 mg (OR: 1.14; 95% CI: 0.66 to 1.91) Tadalafil 20 mg on demand (OR 0.92; 95% CI: 0.52 to 1.56)
Treatment discontinuation	Daily dose (≥6 months)	Philippou et al., 2018 [18]	1 RCT	420 (210 intervention group vs. 210 control group)	RR: 1.12; 95% CI: 0.85 to 1.48; *p* = 0.41
	Daily dose vs. on-demand dose (≥6 months)	Philippou et al., 2018 [18]	3 RCTs	612 (307 daily dose group vs. 305 on-demand dose group)	RR: 1.09; 95% CI: 0.86 to 1.38; *p* = 0.98; I^2^: 0%
Adverse events	Headache	Wang et al., 2014 [21]	6 RCTs	1820 (1205 intervention group vs. 615 control group)	POR: 2.99; 95% CI: 2.22 to 4.04; *p* < 0.00001; I^2^: 0%
	Flushing	Wang et al., 2014 [21]	6 RCTs	1832 (1213 intervention group vs. 619 control group)	POR 4.71; 95% CI: 3.19 to 6.95; *p* < 0.00001; I^2^: 0%
	Dyspepsia	Wang et al., 2014 [21]	4 RCTs	1470 (980 intervention group vs. 490 control group)	POR 4.71; 95% CI: 3.19 to 6.95; *p* < 0.00001; I^2^: 0%
	Nasal congestion	Wang et al., 2014 [21]	5 RCTs	1709 (1132 intervention group vs. 570 control group)	POR 2.66; 95% CI: 1.85 to 3.84; *p* < 0.00001; I^2^: 0%
	Serious adverse events (on-demand dose) (short-term)	Philippou et al., 2018 [18]	3 RCTs	443 (220 on-demand dose group vs. 203 control group)	RR: 0.32; 95% CI: 0.11 to 0.94; *p* = 0.04; I^2^: 0%
	Serious adverse events (on daily dose vs. on-demand dose) (short term)	Philippou et al., 2018 [18]	1 RCT	282 (139 daily dose group vs. 143 on-demand dose group)	RR: 0.69; 95% CI: 0.12 to 4.04; *p* = 0.68
	Serious adverse events (on daily dose vs. on-demand dose) (long term)	Philippou et al., 2018 [18]	1 RCT	100 (50 daily dose group vs. 50 on-demand dose group)	RR: 3; 95% CI: 0.13 to 71.92

CI: confidence interval; EF: erectile function; I^2^: heterogeneity; IIEF-EF: International Index of Erectile Function–Erectile Function domain scores; MD: mean difference; PDE5-i: phosphodiesterase type 5-inhibitors; POR: Peto odds ratio; RCT: randomized controlled trial; RP: radical prostatectomy; RR: relative risk.

**Table 2:** Quality assessment of the studies included in the present review.

Reference	Study Type	Quality Assessment
Feng et al., 2022 [16]	Systematic review with meta-analysis	Critically low ^a^
Liu et al., 2017 [17]	Systematic review with meta-analysis	Critically low ^a^
Philippou et al., 2018 [18]	Systematic review with meta-analysis	High ^a^
Sari Motlagh et al., 2021 [19]	Systematic review with meta-analysis	Moderate ^a^
Wang et al., 2014 [21]	Systematic review with meta-analysis	Critically low ^a^
Zhang et al., 2026 [23]	Systematic review with meta-analysis	Moderate ^a^
Corona et al., 2025 [28]	Systematic review with meta-analysis	High ^a^
Rho et al., 2022 [29]	Systematic review with meta-analysis	Critically low ^a^
Quistini et al., 2026 [30]	Systematic review with meta-analysis	High ^a^
Ergun et al., 2025 [31]	Systematic review with meta-analysis	High ^a^
Jacob et al., 2026 [32]	Systematic review with meta-analysis	High ^a^
Zhou et al., 2025 [33]	Systematic review with meta-analysis	High ^a^
Senel et al., 2025 [34]	Systematic review with meta-analysis	Moderate ^a^
Pirola et al., 2024 [24]	Systematic review	Critically low ^a^
Jo et al., 2018 [20]	RCT	High RoB ^b^
Montorsi et al., 1997 [25]	RCT	High RoB ^b^
Haahr et al., 2016 [35]	Phase I clinical trial	Critical RoB ^c^
Yiou et al., 2017 [36]	Phase I/II clinical trial	Critical RoB ^c^

RCT: randomized controlled trial and RoB: risk of bias. ^a^ methodological quality assessed with Methodological Quality of Systematic Reviews-2 [AMSTAR-2, http://www.amstar.ca (accessed on 16 May 2026)] [12]; ^b^ methodological quality assessed with revised tool for risk of bias [rRoB 2, https://www.riskofbias.info/welcome/rob-2-0-tool (accessed on 16 May 2026)] [13]; and ^c^ methodological quality assessed with risk of bias in non-randomized studies of intervention (ROBINS-I) [14].

**Table 3:** Proposed clinical roadmap for early penile rehabilitation following nerve-sparing radical prostatectomy.

Step/Timing	Patient Stratification and Assessment	Suggested Early Treatment Strategy	Pathophysiological and Clinical Rationale
Step 1: Baseline stratification (immediate post-operative phase)	Assess surgical preservation details and patient baseline status. Bilateral nerve-sparing: Highest success triad (younger age, high baseline EF). Unilateral nerve-sparing: Degraded recovery profile; requires aggressive multi-modal attention.	Immediate initiation strategy: Schedule a patient-centered baseline consultation to establish early proactive rehabilitation goals instead of a reactive “wait-and-see” approach.	Identifies candidates highly responsive to first-line therapies vs. those who may require accelerated progression to advanced treatments.
Step 2: First-line intervention (initiation within 1–3 months post-RP)	All eligible patients undergoing nerve-sparing radical prostatectomy with no medical contraindications.	Pharmacological monotherapy: Regular daily or nightly dosing of PDE5 inhibitors (e.g., sildenafil or tadalafil).	Prevents cyclic GMP degradation and amplifies nitric oxide-mediated relaxation. Dosing aims to maintain chronic corporeal oxygenation, counteracting tissue hypoxia during nerve neuropraxia. Immediate post-op initiation significantly improves long-term EF recovery compared to delayed therapy.
Step 3: Supportive multi-modal add-ons (early phase integration)	Optimal for bilateral or unilateral nerve-sparing cohorts to prevent tissue structural collapse during the window of nerve recovery.	Combination therapy integration: Combine scheduled PDE5 inhibitors with VEDs and PFMT.	Pairing modalities with complementary mechanisms achieves synergistic effects. VED utilizes mechanical negative pressure to draw blood into the corpora, stretching tissue to limit structural retraction and progressive fibrosis. PFMT strengthens the bulbocavernosus and ischiocavernosus muscles to optimize the veno-occlusive mechanism.
Step 4: Refractory evaluation and advanced care (assessed if non-responsive to conservative steps)	Patients who remain refractory to non-invasive options show severe cavernous fibrosis or experience significant relational/psychosocial distress.	Second and third-line transition: Shift to intracavernosal alprostadil injections or pursue early referral for PP implantation.	Prostaglandins entirely bypass compromised neural pathways by increasing intracellular cAMP to induce nerve-independent smooth muscle relaxation. PP structurally replaces dysfunctional tissue with inflatable cylinders, completely bypassing damaged neurovascular infrastructure to ensure immediate rigidity with exceptionally high satisfaction rates independent of pelvic surgery history.
Investigational annex (parallel or complementary path)	Highly selected patients enrolled in standardized clinical trials or specialized protocols.	Regenerative therapy protocols: Li-ESWT, PRP, or SC interventions.	Aims to alter the underlying tissue microenvironment. Li-ESWT triggers angiogenic factor release and recruits progenitor cells; PRP activates cellular proliferation and tissue remodeling; SC therapy utilizes paracrine signaling to accelerate neural/microvascular repair and inhibit pro-fibrotic cascades.

cAMP: cyclic adenosine mono-phospate; EF: erectile function; GMP: guanosine mono-phosphate; Li-ESWT: low-intensity extracorporeal shockwave therapy; PDE5: phosphodiesterase type 5; PFMT: pelvic floor muscle training; PP: penile prosthesis; PRP: platelet-rich plasma; RP: radical prostatectomy; SC: stem cell; VED: vacuum erectile device.

## Data Availability

No new data were created or analyzed in this study. Data sharing is not applicable to this article.

## Abbreviations

The following abbreviations are used in this manuscript:

AMSTAR-2	Assessing the Methodological Quality of Systematic Reviews 2
CI	Confidence interval
ED	Erectile dysfunction
EF	Erectile function
HR	Hazard ratio
IIEF	International index of erectile function
IIEF-5	International index of erectile function-5 items
IIEF-EF	International index of erectile function–erectile function
IPP	Inflatable penile prosthesis
Li-ESWT	Low-intensity extracorporeal shock wave therapy
MD	Mean difference
OR	Odds ratio
PDE5-i	Phosphodiesterase type 5-inhibitors
PFMT	Pelvic floor muscle training
PICO	Population, intervention, comparison, outcome
POR	Peto odds ratio
PP	Penile prosthesis
PR	Penile rehabilitation
PRIOR	Preferred reporting items for overviews of reviews
PRP	Platelet-rich plasma
PSV	Peak systolic velocity
RARP	Robot-assisted radical prostatectomy
RCT	Randomized controlled trial
ROBINS-I	Risk of bias in non-randomized studies of intervention
RP	Radical prostatectomy
RR	Relative risk
rROB 2	Revised tool for risk of bias
SC	Stem cell
SMD	Standard mean difference
VED	Vacuum erectile device
